# Assessment of heat-killed *E. coli* expressing Chikungunya virus E2 protein as a candidate vaccine for dual protection against Chikungunya virus and *E. coli*

**DOI:** 10.3389/fimmu.2024.1500622

**Published:** 2025-01-07

**Authors:** Surajit Patra, Virendra Gajbhiye, Yogesh A. Karpe

**Affiliations:** ^1^ Nanobioscience Group, Agharkar Research Institute, Pune, India; ^2^ Savitribai Phule Pune University, Pune, India

**Keywords:** CHIKV, E2 protein, *E. coli*, heat-killed, vaccine

## Abstract

The Chikungunya virus (CHIKV) is a mosquito-borne virus with a long history of recurring epidemics transmitted through *Aedes* mosquitoes. The rapid spread of CHIKV has intensified the need for potent vaccines. Escherichia coli (*E.coli*), a vital part of human gut microbiota, is utilized in recombinant DNA technology for cloning. However, its high adaptability can lead to severe infections in humans. This study aimed to develop the candidate dual vaccine against CHIKV and *E. coli.* For this, we expressed the CHIKV E2 protein in the *E. coli* Rosetta Bl21 cells and the protein expression was confirmed by western blotting. The IgG immune response of the candidate vaccine was determined against CHIKV and *E. coli* by ELISA. Further, the potential of antibodies to neutralize CHIKV was evaluated via Tissue Culture Infectious Dose 50 (TCID50). We observed that cells expressing E2 protein with alum immunized mice serum showed a five-fold higher IgG immune response against CHIKV, compared to control cells. The CHIKV neutralization assay results showed a two-fold decrease in CHIKV TCID50 value after 12 hours and a three-fold reduction after 120 hours. Similarly, the vaccine formulation also elicited a significantly higher IgG immune response against *E. coli*. The results suggested that expressing CHIKV E2 protein in *E. coli* is a potential approach for generating an IgG immune response against CHIKV and *E. coli* both. This study proposes a faster, safer, and cost-effective recombinant protein-based vaccine development.

## Graphical Abstract

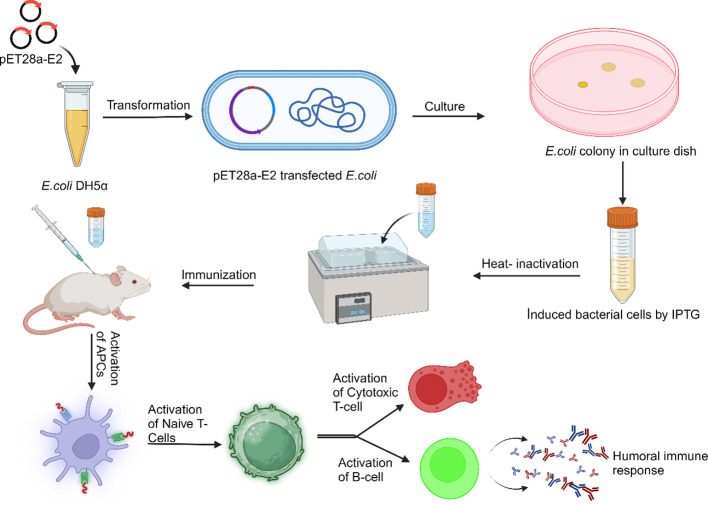

## 1 Introduction

The chikungunya virus (CHIKV) is a positive-sense single-stranded RNA virus of the genus Alphavirus and family Togaviridae. CHIKV has a long history of recurring epidemics (1). CHIKV is primarily transmitted through *Aedes* mosquitoes, specifically *Aedes aegypti* and *Aedes albopictus*, which act as vectors for the virus. Regional epidemics of CHIKV occurred mainly in Africa and Southeast Asia after its identification (2–4). CHIKV infection has an initial stage lasting 2-7 days and is characterized by increased body temperature, vomiting, diarrhea, and joint pain. Post-acute symptoms persist for up to 21 days, and a chronic or arthritic phase may occur in elderly individuals caused by viral encephalitis (1) (2). During the 2005-2007 epidemics in the Indian Ocean islands and India, there were recorded cases of deaths, viral encephalitis, and newborn illnesses connected with CHIKV (3). The length of the CHIKV genome is ~12 kb, including two untranslated regions known as 5’UTR and 3’UTR. CHIKV genome also contains two open reading frames (ORFs) separated by a noncoding junction. The 5’ORF is translated from the genomic RNA (gRNA) that encodes the non-structural polyprotein (P1234) and is later divided into distinct non-structural proteins nsP1 to 4. The 3’ORF translates a positive-sense sub-genomic mRNA (sgRNA) into several structural proteins, including envelope 1 (E1), 6K, envelope 3 (E3), envelope 2 (E2), and capsid (C) (4–7). The rapid spread of CHIKV has increased, and it has become a global pathogen. Being a major public health threat, there is an urgent need for an efficient vaccine. Live-attenuated vaccines are the weaken form or non-virulent form of the virus, reducing its ability to cause illness; similarly, inactivated vaccines render it inactive using chemicals, heat, or UV radiation. These strategies stimulate a robust immune response while ensuring safety and efficacy concerns (8). Subunit vaccines utilize specific virus components, like proteins or peptides, to trigger an immune response, thereby reducing side effects and avoiding total exposure to the virus (9). Vector-based vaccinations use non-pathogenic viruses or bacteria to deliver viral antigens into cells, mimicking a genuine infection to stimulate a potent immune response. Progress in vaccine candidates against chikungunya relies on continuous research and development. Recent advancements have qualified specific candidates for clinical trials, emphasizing the need for diverse immunization strategies (10). The FDA recently authorized Valneva’s single-shot live-attenuated chikungunya vaccine, Ixchiq/VLA1553, on November 9, 2023. This is the only licensed human vaccine against CHIKV; however, several CHIKV candidate vaccines are in clinical trials (11). The lack of long-term monitoring in chikungunya-prone regions such as Africa, Latin America, and Southeast Asia, along with intermittent transmission and unpredictable outbreaks, hinders randomized controlled efficacy studies. This has led authorities to explore alternative data collection methods for developing and authorizing CHIKV vaccines and medical devices (12, 13). The CHIKV E2 surface protein is used in vaccine preparation because the E2 protein is the primary target of neutralizing antibodies, facilitating the virus’s binding to cell membrane receptors and attachment factors. The E2 glycoprotein binds to the Mxra8 membrane receptor, triggering clathrin-mediated endocytosis of CHIKV. Infected individuals develop antibodies that target the E2 protein, which monoclonal antibodies can neutralize. In adult and neonatal mice, anti-E2 antibodies from recovering patients can reduce or eliminate CHIKV infection (4, 5, 7).

*Escherichia coli* is a versatile bacterium that is a crucial part of human gut microbiota and is being used in recombinant DNA technology for cloning. However, due to its high adaptability, *E. coli* can cause severe infections. Many strains of *E. coli* use virulence factors that affect various cellular functions, leading to intestinal and extraintestinal diseases. Despite its potential danger, *E. coli* remains a valuable laboratory organism (14). *E. coli* bacteria colonize human infants’ gastrointestinal tracts after birth, coexisting in a symbiotic state. They rarely cause illness unless the immune system weakens or regular gastrointestinal tract barriers are breached. The mucous layer of the mammalian colon is the habitat for commensal *E. coli*, the predominant facultative anaerobe of the human gut microbiota. The mechanisms behind this symbiotic relationship remain unclear, but a hypothesis suggests it uses gluconate in the colon (15). Some aggressive *E. coli* strains have acquired characteristics that allow them to adapt to new environments and cause a broader range of diseases. The pathotypes that can infect healthy individuals are limited to the most efficient combinations of virulence factors. These pathotypes can lead to enteric/diarrheal disease, urinary tract infections (UTIs), or sepsis/meningitis. The six distinct families of intestinal pathogens are Enteropathogenic *E. coli* (EPEC), Enterohaemorrhagic *E. coli* (EHEC), Enterotoxigenic *E. coli* (ETEC), Enteroaggregative *E. coli* (EAEC), Enteroinvasive *E. coli* (EIEC), and Diffusely Adherent *E. coli* (DAEC) (16). Urinary tract infections and sepsis are primarily caused by Extraintestinal pathogenic *E. coli* (ExPEC), a multidrug-resistant strain, leading to treatment failure and hospitalization costs. Adequate immunization could reduce illness and mortality rates, potentially decreasing the bacteria’s prevalence in both healthy and sick populations. Genomic analysis revealed the gene sinH is prevalent in several invasive ExPEC phylogroups (17). ETEC is a genetically diverse pathogen causing millions of symptomatic infections annually, primarily affecting children in low and middle-income countries. Initially discovered 40 years ago, ETEC contributes to the death rate among children under five and causes severe diarrhea. Developing effective vaccinations to prevent ETEC infections remains crucial, but the inherent genetic variability of *E. coli* poses a significant challenge (18). *E. coli* strain O157:H7, a zoonotic enteric pathogen, has the potential to induce life-threatening hemolytic uremic syndrome (HUS) because of Shiga toxins (Stx). Additionally, it can lead to severe infections that result in chronic renal failure in children. Hemorrhagic colitis and diarrhea are two commonly recognized symptoms of infection (19). *E. coli* pathogenic strains cause damage to mucosal surfaces through a sequential pathogenesis process, including colonization, evasion of the host’s immune system, proliferation, and harm. While most *E. coli* strains remain outside cells, EIEC is a genuine intracellular pathogen capable of invading and replicating within macrophages and epithelial cells. Some strains can enter epithelial cells but do not replicate inside them (14). Developing vaccines for *E. coli* is complex due to the diverse strains and virulence levels. Researchers aim to create safe and effective vaccines that provide broad protection against various strains by focusing on common targets, shared antigens, and genetic diversity, ensuring a comprehensive strategy to address the diverse pathogenic properties of these strains. This work mainly focuses on preparing an *E. coli*-based recombinant protein vaccine to protect against viral (CHIKV) and bacterial (*E. coli*) infection.

## 2 Materials and methods

### 2.1 Animal ethics statement

The BALB/c mice were obtained from the in-house animal facility at ARI. All animal protocols were approved by the ARI-Institutional Animal Ethics Committee (IAEC).

### 2.2 Virus and cell line

The Chikungunya virus strain IND-06-AP3 (GenBank accession no: EF027134.1) was obtained from NIV, Pune. The Vero C1008 cell line was obtained from NCCS, Pune. *E. coli* DH5α and Rosetta Bl21 were procured from Novagen.

### 2.3 Preparation of plasmid for protein expression

The pET28a-E2 was developed previously in our lab. We modified it and removed the 6-HIS Tag from the T7 region by Gibson assembly cloning, and all primers were designed through NEBuilder Assembly Toolv2.6.1. Primer sequences:-

(E2F: ggagctcgaattcggaGCAGCATATTAGGCTAAGC;

E2R: taagaaggagatataccatCCATACTTAGCTCACTGTCC;

pET28aF:ATGGTATATCTCCTTCTTAAAGTTAAAC;

pEt28aR: TCCGAATTCGAGCTCCGTC).

### 2.4 Preparation of induced *E. coli* for mouse immunization

Plasmid pET28a-E2 was transformed into *E. coli* Rosetta Bl21 cells. The cells were grown in Luria-Bertani broth (LB) overnight at 37°C and 160 rpm. When the bacterial culture OD reached 0.4, CHIKV E2 protein expression in Rosetta Bl21 cells was induced using 1mM IPTG treatment and a continuous culture at 20°C and 160 rpm overnight. After the induction, the bacterial cells were centrifuged at 5000 rpm for eight minutes at 4°C. The cell pellets were washed three times in PBS at 5000 rpm for eight minutes at 4°C and re-suspended in 5 ml PBS (19).

### 2.5 Western blot analysis

Both *E. coli* Rosetta Bl21 expressing CHIKV E2 protein and control Rosetta Bl21 were mixed with Laemlli’s buffer, and cells were incubated for 10 mins at 95°C in a dry bath before being subjected to SDS-PAGE and transferred to a PVDF membrane (Bio-Rad). The membranes were blocked in Western blocker solution (sigma) for 2 hours at room temperature. The membranes were washed three times with PBS containing 0.1% Tween 20 (PBST) before incubating with mouse IgG primary antibody against CHIKV E2 protein (in-house, dilution used 1:1000) for 2 hours at room temperature. The membranes were washed thrice with PBST (20). After washing, the membranes were probed with an anti-mouse IgG HRP conjugated antibody (secondary antibody, dilution used 1:15000) for 1 hour at room temperature. The membrane was developed using a chromogenic substrate called ECL (sigma) and visualized in ChemiDoc (Bio-Rad).

### 2.6 Preparation of heat-killed *E. coli* and mouse immunization

Bacterial cell suspensions were heated at 70°C for one hour to prepare heat-killed bacteria for vaccination. The bacterial death was confirmed by LA platting of both heat-killed bacteria for incubating one week at 37°C (19). For the immunization study, BALB/c mice (male and female, aged 5-6 weeks) were selected and divided into three groups according to **Supplementary Table S1**. The mice were immunized subcutaneously (100 μl) by 1×10^8^ heat-killed bacteria (according to the **Supplementary Table S1**).

### 2.7 Viral triter determination by TCID50

For the TCID50 assay, 10^4^ Vero cells were seeded in each well of 96 well plates and kept overnight at 37°C, 5% CO_2_, and 90% humidity. The next day, culture media was removed, and cells were washed with incomplete MEM media. Initially, the CHIKV stock was (100 μl) serially diluted ten times in incomplete MEM media, and serial dilution was done inversely from 10^–1^ to 10^–10^. After that, 100 μl of serially diluted virus solution was added in each well, and for each same concentration, eight replicates were taken from 10^–3^ to 10^–10^ and incubated at 37°C, 5% CO_2_, and 90% humidity for one and half hour. After completion of the incubation period, incomplete MEM media was removed from each well and washed once with PBS to remove the uninfected virus from each well. Then 200 μl of complete MEM media (5% FBS) was added to each well and incubated at 37°C, 5% CO_2_, and 90% humidity for five days. After five days, cells were examined under a microscope for any cytopathic consequences, such as cell rounding or detachment, resulting from viral infection (21). Wells exhibiting cytopathic effects (CPE) have been classified as being infected. The data was collected by microscopic identification of the virus infection on Vero cells in each well. According to the data, TCID50 of CHIKV was calculated at every time point by the Spearman & Kärber algorithm as described in Hierholzer & Killington (1996), Virology Methods Manual, p. 374.

### 2.8 ELISA to determine IgG immune response against CHIKV

Serum levels of IgG immune response against CHIKV were determined by using an IgG(Total) Uncoated ELISA Kit with Plates (Catalog # 88-50400-86; Invitrogen). To determine the IgG immune response against CHIKV, 10^–1^ CHIKV (Stock 2.37×10^8^ TCID50/ml) in 100 μl of coating buffer was coated in each well of 96 well ELISA plate (corning) and kept the ELISA plate at 4°C overnight for CHIKV coating. The next day, plates were washed with PBST (PBS+0.05% Tween 20) in a plate washer two times in continuous rocking, after that the plates were blocked with 250 μl of 5% fat-free milk powder in PBS for 2 hours. After that, plates were washed with PBST (PBS+0.05% Tween 20) in a plate washer three times. All the serum samples (primary antibody) were diluted (1:100) in assay buffer (Supplied in IgG assay kit, Invitrogen), and 100 μl of diluted sample was added in each well and incubated for two and half hours at room temperature on a rocker. After that, the ELISA plate was washed with PBST four times in a plate washer, and 50 μl of secondary IgG antibody (Anti-mouse IgG secondary antibody with HRP; Invitrogen) was added to each well (diluted 1:250 according to manufacturer protocol) and incubated for two hours at room temperature on rocker. After incubation of the secondary antibody, ELISA plates were washed with PBST five times. TMB substrate was added 100 μl in each well and incubated at room temperature for 15 minutes; after that, 100 μl of 1M ortho-phosphoric acid solution was added in each well to stop the HRP and TMB substrate reaction, and absorbance was measured at 450 nm in a plate reader. A serum sample was categorized as reactive if its optical density (OD) value was equal to or above three times the average OD value (ELISA threshold) of preimmune sera (negative control). The antibody titer specific to CHIKV was calculated by calculating the reciprocal of the highest dilution that produced an absorbance equal to or greater than the ELISA threshold (22).

### 2.9 ELISA to determine IgG immune response against *E. coli* Rosetta Bl21

Serum levels IgG immune response against E.coli Rosetta Bl21 was determined by using IgG(Total) Uncoated ELISA Kit with Plates (Catalog # 88-50400-86; Invitrogen). 1×10^8^ *E. coli* Rosetta Bl21 was coated in 100 μl coating buffer in each well of 96 well ELISA plates, and the ELISA plate was kept at 4°C overnight for E. coli Rosetta Bl21 coating (19). The rest of the process followed as described earlier in section 2.8.

### 2.10 ELISA to determine total IgG1, and IgG2b immune response

Serum levels of total IgG1, and IgG2b in mice were analyzed using an IgG1 mouse Uncoated ELISA Kit with Plates (Catalog # 88-50410-22; Invitrogen), and an IgG2b mouse Uncoated ELISA Kit with Plates (Catalog # 88-50430-22; Invitrogen), respectively. The capture antibody was diluted in 1X coating buffer (1:250) and added in 100 μl in each well of 96 well ELISA plates (corning), and the ELISA plate was kept at 4°C overnight for coating of capture antibody. The next day, plates were washed with PBST (PBS+0.05% Tween 20) in a plate washer two times in continuous rocking, after that in continuous rocking, the plates were blocked with 250 μl of 2X assay buffer for 2 hours. After that, plates were washed with PBST in a plate washer two times. All the serum samples (primary antibody) were diluted (1:50000 for IgG1 and IgG2b) in assay buffer (Supplied in assay kit, Invitrogen), and 100 ml of diluted sample was added in each well and incubated for two hours at room temperature on a rocker. After that, the ELISA plate was washed with PBST two times in a plate washer, and 50 μl of secondary IgG antibody (Anti-mouse IgG secondary antibody with HRP; Invitrogen) was added to each well (diluted 1:250 according to manufacturer protocol) and incubated for two hours at room temperature on rocker. After incubation of the secondary antibody, ELISA plates were washed with PBST four times. TMB substrate was added 100 μl in each well and incubated at room temperature for 15 minutes; after that, 100 μl of 1M ortho-phosphoric acid solution was added in each well to stop the HRP and TMB substrate reaction, and absorbance was measured at 450 nm in a plate reader.

### 2.11 *In vitro* viral neutralization assay by TCID50

*In vitro*, viral neutralization was determined by TCID50 assay. For this, 10^4^ Vero cells were seeded in each well of 96 well plates and kept overnight at 37°C, 5% CO_2_, and 90% humidity. For neutralization, the mouse serum was inactivated (collected after 58 days of mouse immunization) in a water bath at 56°C for 1 hour. The main stock of CHIKV concentration was 2.37×10^8^ TCID50/ml. Then, 100 μl of this CHIKV stock was taken in each of five different centrifuge tubes. In 1st and 2nd tubes, 1 and 2 μl of E. coli Rosetta Bl21 expressing E2 with alum-injected mouse serum (pooled) was added, respectively. In 3rd and 4th tubes, 1 and 2 μl of E. coli Rosetta Bl21 with alum-injected mouse serum (pooled) was added, respectively. Tube-5 was taken as positive CHIKV control. All these tubes were incubated at 37°C for 1 hour. Then, each tube was serially diluted ten times (10^–1^ to 10^–10^ of CHIKV) by incomplete MEM media. The culture media from 96 well plates was removed and washed with incomplete MEM media. After that, 100 μl of every serially diluted virus solution was added to a well containing Vero cells. Each concentration had eight replicates, and these were incubated at 37°C, 5% CO_2_, and 90% humidity for one and a half hours (23). After completion of the incubation period, incomplete media was removed from each well, and plates were washed once with PBS to remove the uninfected virus from each well. Then 200 μl of complete MEM media (5% FBS) was added to each well, and plates were incubated at 37°C, 5% CO_2_, and 90% humidity for five-days and a half days. Using a microscope, cells were examined after 12, 24, 48, 72, 96, and 120 hours for any cytopathic effects, such as cell rounding or detachment, resulting from viral infection. Wells exhibiting cytopathic effects (CPE) were classified as infected (21). Wells exhibiting cytopathic effects (CPE) have been classified as being infected. The data was collected by microscopic identification of the virus infection on Vero cells in each well. According to the data, TCID50 of CHIKV was calculated at every time point by the Spearman & Kärber algorithm as described in Hierholzer & Killington (1996), Virology Methods Manual, p. 374.

### 2.12 Statistical analysis

Each experiment was performed three times, and the data were thereafter given as means ± standard deviation (SD). The statistical analysis was performed using the GraphPad Prism software version 8.0.1.244. A two-way analysis of variance (ANOVA) with Bonferroni posttests was applied to evaluate the statistical significance. An observed p-value < 0.05 was considered statistically significant.

## 3 Results

**Figure 1:**
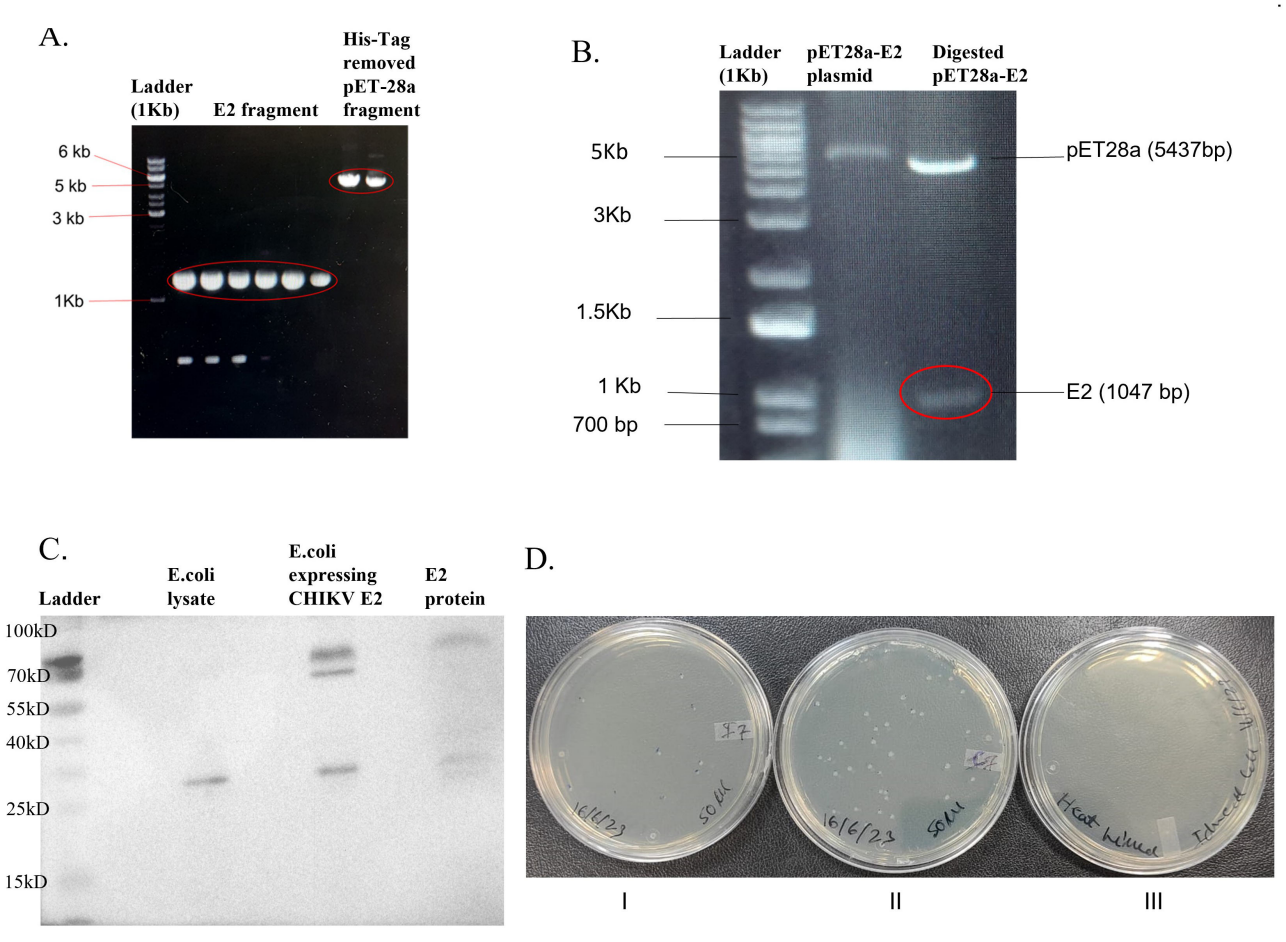
Modified pET28a-E2 plasmid and pET28a-E2 transform *E. coli* Rosetta BL21 expressing CHIKV E2 protein. **(A)** Gel image of E2 fragments and modified pET28a plasmid fragments after PCR; **(B)** Agarose gel image showing XbaI and NcoI digested pET28a-E2 plasmid; **(C)** Protein expression of modified pET28-E2 transform *E. coli* Rosetta cell Bl21 cells; and **(D)**, (I). *E. coli* Rosetta bl21 cells expressing CHIKV E2 protein, (II). *E. coli* Rosetta bl21 cells (Control) and (III). Heat-killed of bacteria.

The pET28a-E2 plasmid was modified to remove 6-His-Tag by Gibson assembly cloning (**Figure 1A**), and the pET28a-E2 plasmid was transformed into *E. coli* DH5α bacterial cells. Clones were confirmed by restriction digestion (**Figure 1B**) and DNA sequencing. The pET28a-E2 plasmid was transformed into *E. coli* Rosetta Bl21 cells and cells were induced by 1mM IPTG for CHIKV E2 protein expression. A western blotting experiment was carried out to confirm CHIKV E2 protein expression in Rosetta Bl21 cells. Western blotting results confirmed CHIKV E2 protein expression with a band of expected molecular weight 47 kDa (**Figure 1C**). Purified CHIKV E2 protein showed protein bands at the same range of 57 kDa, but the control *E. coli* bacteria did not do so.

CFU method was used to calculate the bacterial number (**Figures 1DI–III**). The *E. coli* Rosetta Bl21 expressing CHIKV E2 protein bacteria concentration was 1.05×10^10^ CFU/ml (**Figure 1DI**), and the *E. coli* Rosetta Bl21 (control strain) concentration was 2.2×10^11^ CFU/ml (**Figure 1DII**). Bacterial cells were killed by heating at 70°C for 1 hour. Bacterial cell death was confirmed by platting on an LA plate and incubated for one week at 37°C. No bacterial colonies were observed on an LA plate (**Figure 1DIII**).

The *E. coli* Rosetta Bl21 expressing CHIKV E2 protein and control *E. coli* Rosetta Bl21 were used for mouse immunization. For mouse immunization, 1×10^8^ bacterial cells were used for both *E. coli* Rosetta Bl21 expressing CHIKV E2 protein and *E. coli* Rosetta Bl21 strains.

The mice were divided into three groups. The first group received *E. coli* Rosetta Bl21 expressing E2 protein with alum. The second group received *E. coli* Rosetta Bl21 bacteria (control) with alum. The ratio of bacteria to alum was 1:1. The third group received *E. coli* Rosetta Bl21 expressing E2 protein without alum. All groups received 1×10^8^ bacteria subcutaneously.

A thirty-day gap was kept between the two doses of immunization. Then, blood was collected by bleeding through the retro-orbital route, and serum was isolated and stored at -20°C for further use. ELISA test was performed to measure the mouse IgG immune response against CHIKV and *E. coli*. CHIKV was coated in an ELISA plate for determining the mouse IgG immune response against CHIKV.

**Figure 2:**
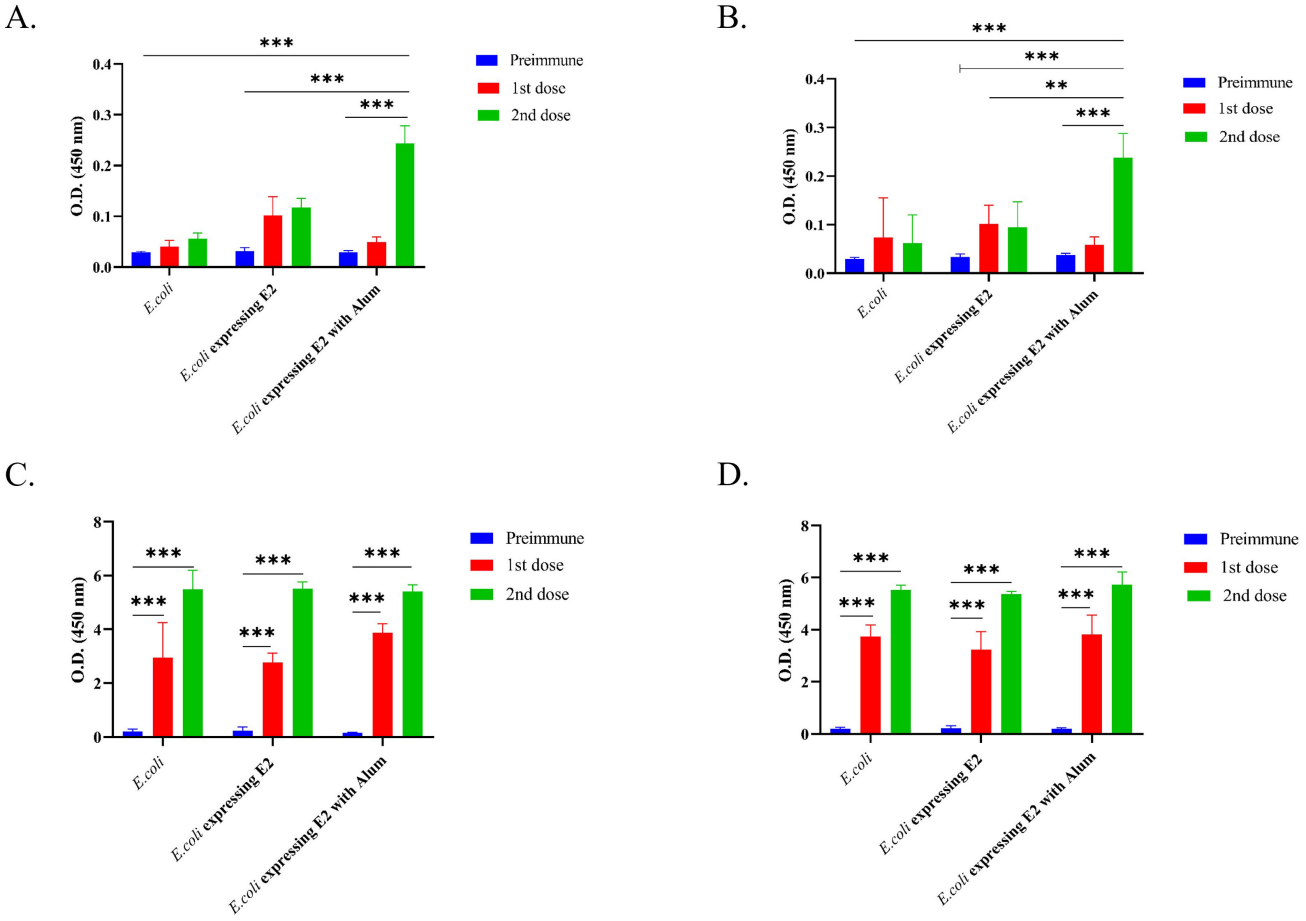
IgG response against different vaccine formulations and control, **(A)** Male BALB/c mice IgG immune response against CHIKV, **(B)** Female BALB/c mice IgG immune response against CHIKV, **(C)** Male BALB/c mice IgG immune response against *E. coli* and **(D)** Female BALB/c mice IgG immune response against *E. coli* (2way ANOVA was used for statistical analysis; ***p<0.001; **p=0.002).

We observed that *E. coli* Rosetta Bl21 expressing E2 protein with alum (OD value 0.244 after 2^nd^ dose) showed a two-fold higher IgG immune response against CHIKV in comparison to *E. coli* Rosetta Bl21 expressing E2 protein without alum (OD value 0.118 after 2^nd^ dose) (**Figure 2A**) in male mice. *E. coli* Rosetta Bl21 expressing E2 protein with alum showed a five-fold higher IgG immune response than control group *E. coli* with alum (OD value 0.056 after 2^nd^ dose) in male mice (**Figure 2A**).

Similar results were observed for female BALB/c mice (**Figure 2B**), wherein *E. coli* Rosetta Bl21 expressing E2 protein with alum (OD value 0.238 after 2^nd^ dose) showed more than two-fold higher IgG immune response against CHIKV in comparison to *E. coli* Rosetta Bl21 expressing E2 protein without alum (OD value 0.095 after 2^nd^ dose), and four-fold higher IgG immune response than control group *E. coli* (OD value 0.062 after 2^nd^ dose). The results confirmed that the E2 protein-expressing strain with alum exhibited a significantly higher IgG immune response against CHIKV (p<0.005) than the E2 protein-expressing strain without alum and the control *E. coli* strain.

*E. coli* Rosetta Bl21 was coated in an ELISA plate to determine the mouse IgG response against *E. coli*. **Figures 2C, D** represent the IgG immune response of different formulations against *E. coli*. A significantly higher IgG triter was observed for all formulations in both male (**Figure 2C**) and female (**Figure 2D**) immunized mice against *E. coli*. E2 protein-expressing strain with alum, E2 protein-expressing strain without alum, and the control *E. coli* strain exhibited OD values of 5.402, 5.517, and 5.5, respectively, after 2^nd^ dose in male mice (**Figure 2C** **).** On the other hand, E2 protein-expressing strain with alum, E2 protein-expressing strain without alum, and the control *E. coli* strain exhibited OD values after 2nd dose of 5.736, 5.37, and 5.538, respectively, after 2nd dose in female mice (**Figure 2D**).

**Figure 3:**
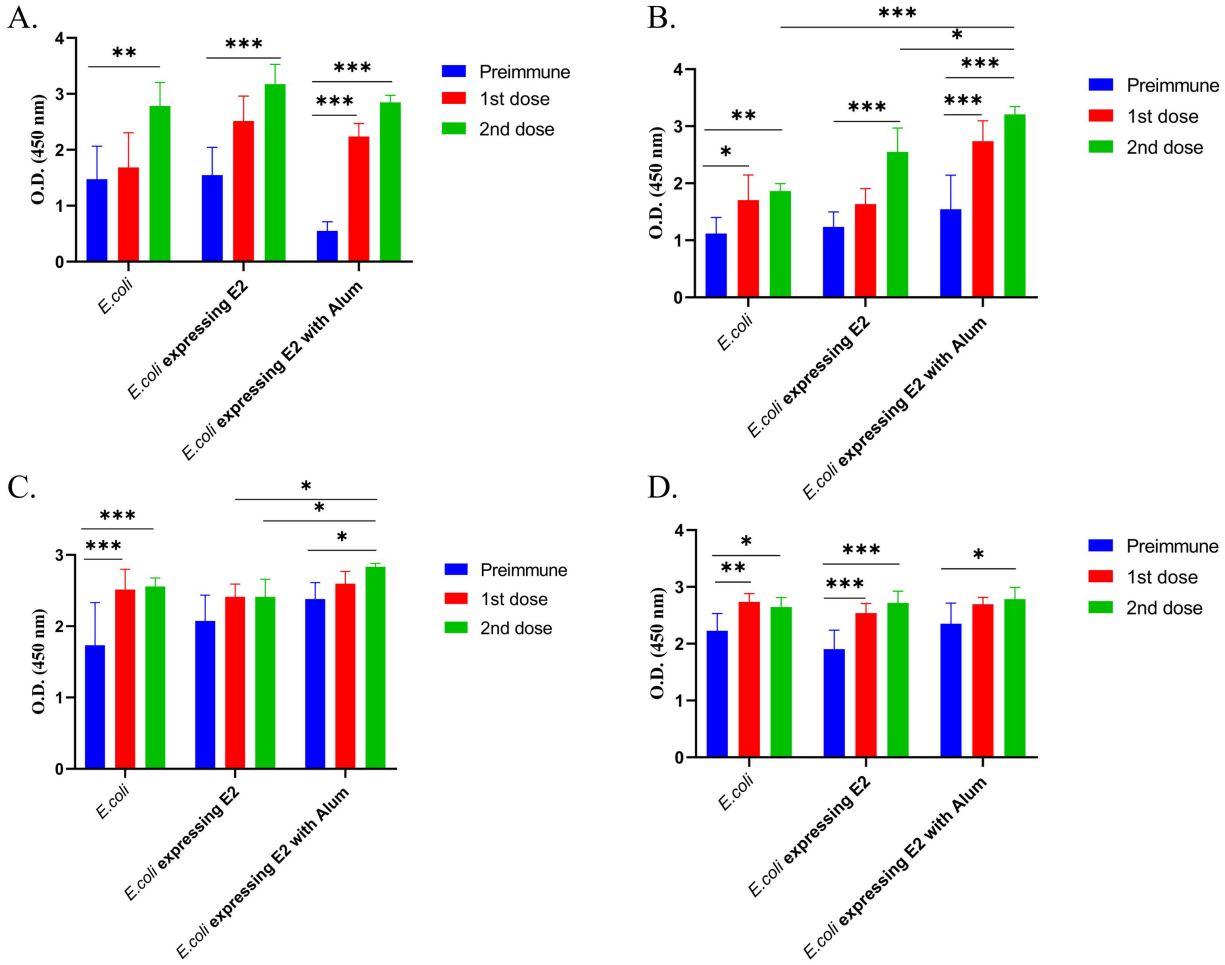
Total IgG1 and IgG2b subtypes response against different vaccine formulations and control, **(A)** Male BALB/c mice Total IgG1 immune response, **(B)** Female BALB/c mice total IgG1 immune response, **(C)** Male BALB/c mice total IgG2b immune response and **(D)** Female BALB/c mice total IgG2b immune response (2way ANOVA was used for statistical analysis; ***p<0.001; **p=0.0021; and *p=0.02).

Anti-mouse IgG1 was coated in an ELISA plate to determine the mouse total IgG1 response. **Figures 3A, B** represent the total IgG1 immune response. A significantly higher IgG1 triter was observed for all formulations in both male (**Figure 3A**) and female (**Figure 3B**) immunized mice. E2 protein-expressing strain with alum, E2 protein-expressing strain without alum, and the control E. coli strain exhibited OD values of 2.85, 3.17, and 2.78, respectively, after 2nd dose in male mice (**Figure 3A**), however the preimmune blood Total exhibited total IgG1 OD values of 0.55, 1.55, and 1.48. Which means that the E2 protein-expressing strain with alum injected mice group showing significant higher IgG1 response then the others groups in male mice. On the other hand, E2 protein-expressing strain with alum, E2 protein-expressing strain without alum, and the control E. coli strain exhibited OD values after 2nd dose of 3.21, 2.5, and 1.86, respectively, in female mice (**Figure 3B**).

We also measured the total IgG2b immune response and observed that E2 protein-expressing strain with alum, E2 protein-expressing strain without alum, and the control *E. coli* strain exhibited OD values of Exhibited OD value 2.83, 2.4 and 2.55 after 2nd dose (**Figure 3C**) in male mice. On the other hand, E2 protein-expressing strain with alum, E2 protein-expressing strain without alum, and the control E. coli strain exhibited OD values after 2nd dose of 2.78, 2.71, and 2.64, respectively, after 2nd dose in female mice (**Figure 3D**).

Pooled serum samples (male and female mice) from E2 protein-expressing strain with alum and controlled strain with alum, immunized mice serums were used for further analysis. CHIKV *in vitro* neutralization assay was performed using TCID50 assay. CHIKV viruses were treated with mouse-immunized serum for one hour at 37°C for neutralization. Then, Vero cells were infected by serum-treated CHIKV viruses with inverse dilution from 10^–1^ to 10^–10^ virus.

**Figure 4:**
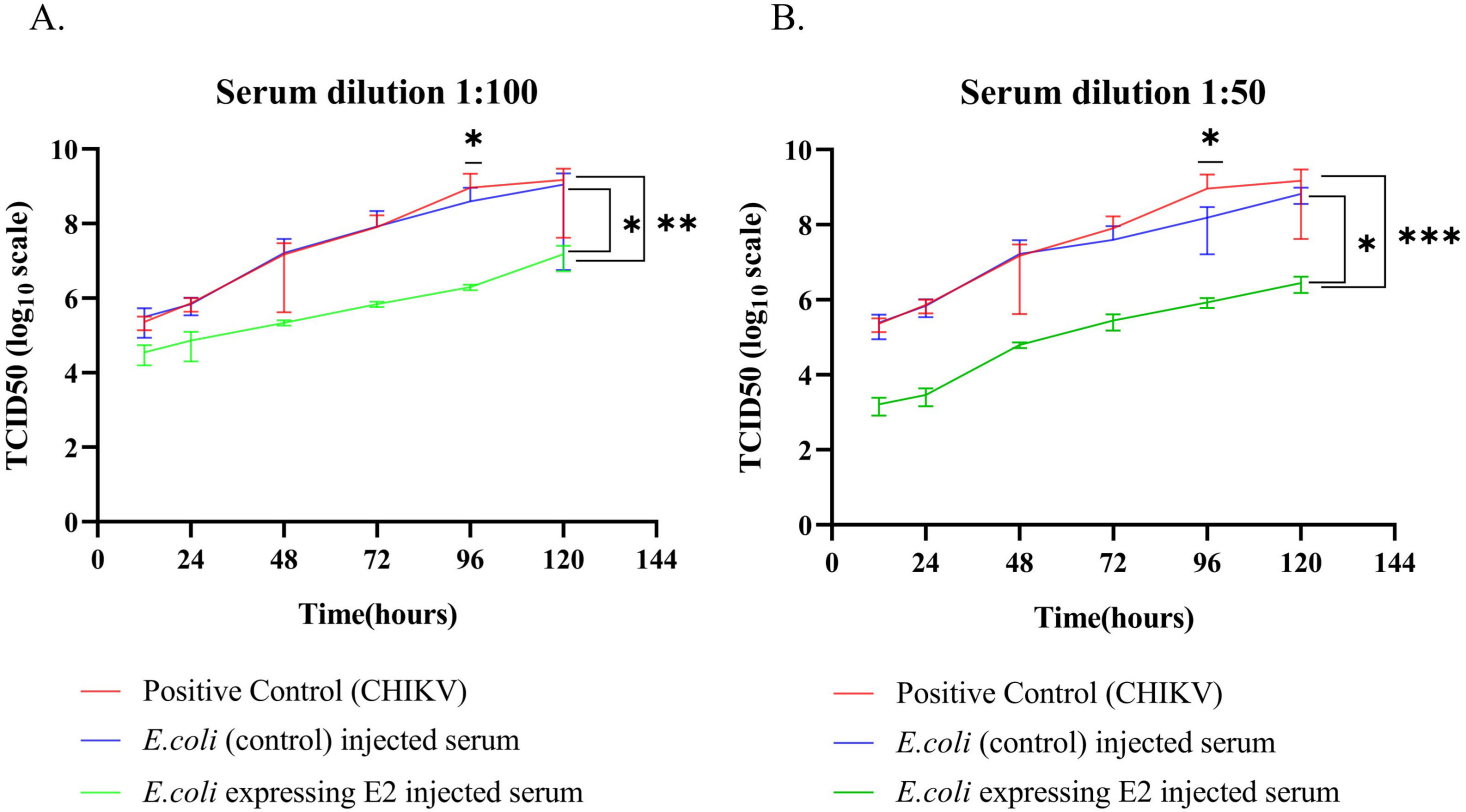
*In vitro* CHIKV neutralization in Vero C1008 cell line **(A)** Serum dilution 1:100 and **(B)** Serum dilution 1:50. (2way ANOVA was used for statistical analysis; *p=0.034; **p=0.001; ***p=0.001).

At serum dilution 1:100, we observed that CHIKV viruses treated with E2 protein-expressing strain with alum-injected mice serum showed a one-fold decrease in TCID50 value at 12 hours from other groups. Further, the same group showed a two-fold reduction in the TCID50 value (**Figure 4A**) after 120 hours from other groups. When the serum dilution was 1:50, there was a two-fold decrease after 12 hours and a three-fold reduction after 120 hours in TCID50 value compared to both *E. coli* Rosetta Bl21 with alum (control) and positive CHIKV infection (**Figure 4B**).

## 4 Discussion

During the ongoing COVID-19 pandemic, the whole world faced a crisis. More than 776 million people were infected, and 7.1 million people died of coronavirus infection (https://data.who.int/dashboards/covid19). Twelve COVID-19 vaccines approved by WHO for emergency were used to generate protection among the world population. Out of the twelve vaccines, two were mRNA-based vaccines, four were non-replicating viral vector-based vaccines, three were inactivated virus-based vaccines, and three were recombinant vaccines (https://covid19.trackvaccines.org/agency/who/). During the pandemic, nucleoside-modified (mRNA and non-replicating viral vector-based vaccines) vaccines came first because of the less time needed to synthesize the vaccine. Synthetic mRNA, a therapeutic component of vaccines, has been used for over 30 years. However, limited research exists on its distribution in the body, cell absorption, translation, and functional lifespan. A recent study found that mRNA can reverse the transcription of vaccine sequences in transfected cells, causing potential safety issues (24).

The continuous presence of NMS-mRNA in the cytoplasm disrupts the removal process, activating natural transposable elements (TEs) and triggering a coordinated innate immune response against foreign genetic material. This can lead to auto-inflammatory and autoimmune disorders and harmful mutations in reverse-transcribed molecules, potentially causing DNA damage. Understanding the intracellular processes triggered by mRNA absorption is crucial for effective vaccine development (24). mRNA vaccines are vital in vaccination campaigns, but they can cause adverse effects due to the pro-inflammatory properties of lipid nanoparticles or mRNA. Further investigation into these mechanisms is needed to ensure safety, build trust, and inform health regulations, despite existing understanding primarily from cell experiments (25). Therefore, in the COVID-19 period, the vaccine was the most critical priority. However, protein-based vaccines are safer to use as vaccines.

Nevertheless, the problem associated with protein-based vaccines is the purification of protein through HPLC, size-based chromatography, and ion-exchanged chromatography. Further, it takes time to optimize protein purification and confirmation. In this study, our findings suggest a faster, safer, and cost-effective recombinant protein-based vaccine preparation technique that can protect against both viral and bacterial infection. The results show that our advanced technique generates an IgG immune response against CHIKV and a robust IgG immune response against *E. coli*. The IgG1 is the primary IgG immunoglobulin in mice. IgG1 is primarily associated with a Th2 based immune response and activate CD8^+^ T-cells. The higher IgG1 immune response shown on E2 protein-expressing strain with alum mice groups. IgG1 can interact with FcγRs on dendritic cells (DCs), leading to T cell activation and cytokine synthesis. IgG1 can bind to FcγRs on dendritic cells, specifically FcγRI and FcγRII. This binding results in the internalization of antigen-IgG complexes, known as immune complexes (ICs). The internalization of immunological complexes by dendritic cells stimulates Fc gamma receptors, thereby augmenting endosomal maturation and lysosomal fusion. This improves antigen processing. DCs process antigens for presentation on major histocompatibility complex (MHC) class II molecules. This presentation activates antigen-specific T cells (26–29). In mice, IgG2b antibodies are mostly associated with a Th1-type immune response that activates CD4^+^ T cells. IgG2b engages with activating Fc receptors on immune cells, particularly FcγRIV, which is essential for triggering a Th1 response (30, 31). The *in vitro* neutralization assay against CHIKV showed three-fold and two-fold viral neutralization against control in 1:50 and 1:100 serum dilution, respectively. Our vaccine preparation method/technique is easy to perform, and there is a minimum chance of post-purification protein miss-folding because recombinant protein is present within the cells.

Another problem with vaccine development against infectious agents like viruses and bacteria is the working person, laboratory leakage, and safety concerns. A biosafety laboratory is mandatory to work against infectious agents, and the cost of preparing and maintaining this laboratory is very high. During the COVID-19 pandemic, the world faced testing of samples and research facilities because of insufficient biosafety laboratory systems. Also, expertise, large-scale production, deployment, and storage were significant challenges (32). The cost associated with biosafety facilities also affects vaccination programs directly. Therefore, our approach of recombinant protein-expressing bacteria as a candidate vaccine can quickly solve this problem. This can be due to fewer biosafety concerns, low-cost production, easy handling, and rapid vaccine production.

Furthermore, this bacteria-expressing viral protein vaccine can be stored at −20°C. Thus, the storage cost can be significantly reduced. This approach can also be used to immunize humans against pathogenic bacteria using non-pathogenic animal bacteria. For example, *Salmonella sp.*, which causes fowl typhoid and is non-pathogenic to humans, can be used for immunization against typhoid and other infectious diseases. Further research in *E. coli*-based recombinant protein vaccines could help prepare vaccines that protect from multiple infections.

## Data Availability

The raw data supporting the conclusions of this article will be made available by the authors, without undue reservation.
