# Supplementary material for: Assessment of heat-killed E. coli expressing Chikungunya virus E2 protein as a candidate vaccine for dual protection against Chikungunya virus and E. coli
Source: Front Immunol. 2025 Jan 7;15:1500622. doi: 10.3389/fimmu.2024.1500622 (PMC11746998; doi:10.3389/fimmu.2024.1500622)
Supplement: Supplementary file 1 [file DataSheet1.docx]

**Table S1: Animal study design for mice immunization**

| **S. No.** | **Group** | **Number of Animals**  **(BALB/c; Age: 5-6 weeks)** | **Subcutaneous injection volume** | **Dosing** | **Bleeding** |
| --- | --- | --- | --- | --- | --- |
| 1 | 1×10^8^ Heat-killed *E. coli* Control+Alum* | 8 for each group  (4 male and 4 female) | 100 μl for each mouse | 1^st^ and 2^nd^  (30 days apart) | Through retro-orbital plexus (50μl).  Preimmune, after 1^st^ and 2^nd^ dose 28 days apart |
| 2 | 1×10^8^ Heat-killed *E. coli* expressing CHIKV E2 |  |  |  |  |
| 3 | 1×10^8^ Heat-killed *E. coli* expressing CHIKV E2 + Alum |  |  |  |  |

***Imject Alum (Thermo Scientific)**

**
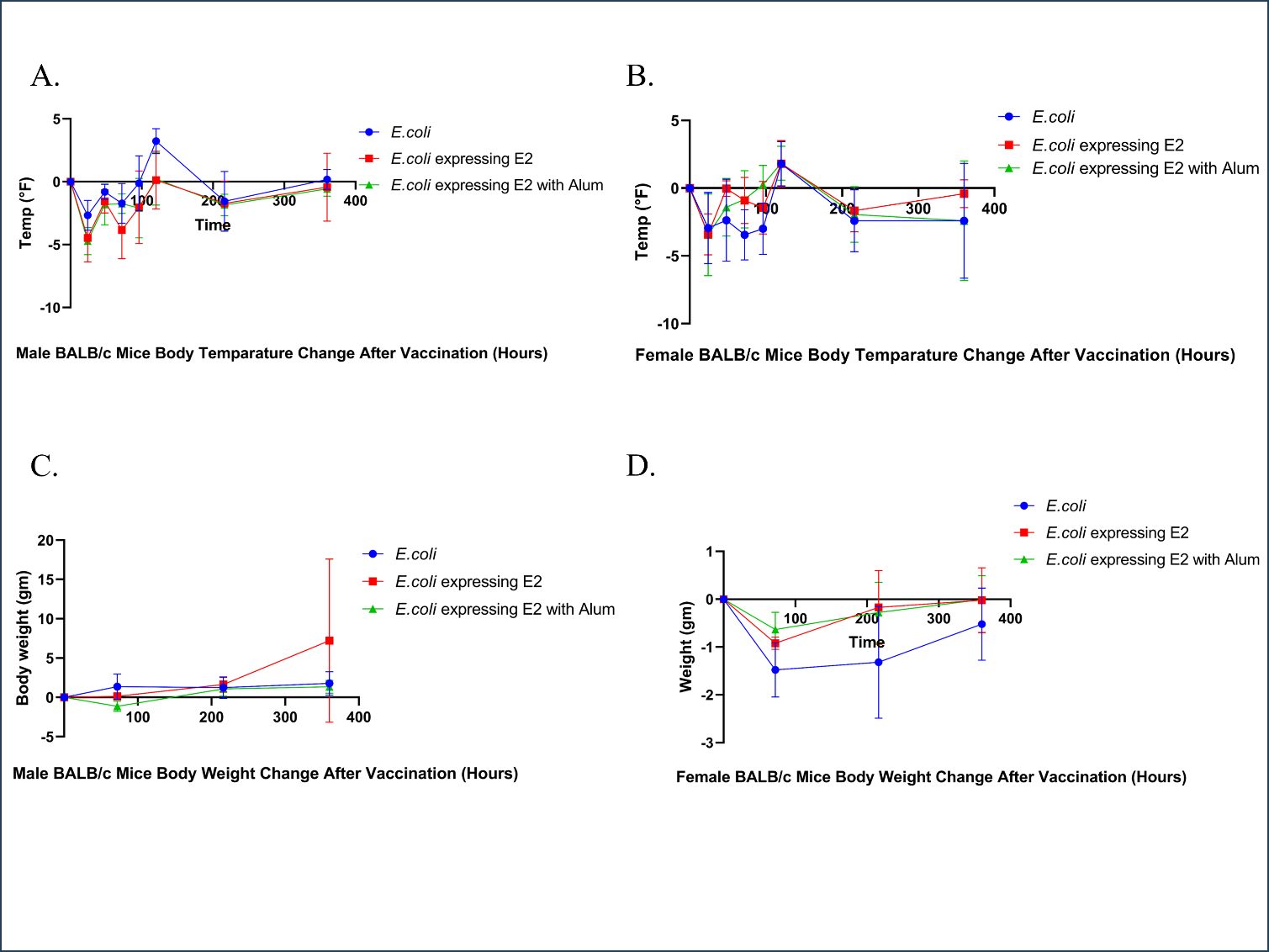
**

**Fig S2: The mice physiological parameter changes after vaccination. A.** Body temperature changes in male BALB/c mice, **B.** Body temperature change in female BALB/c mice, **C.** The male BALB/c mice body weight changed, and **D.** Female BALB/c mice body weight changed.


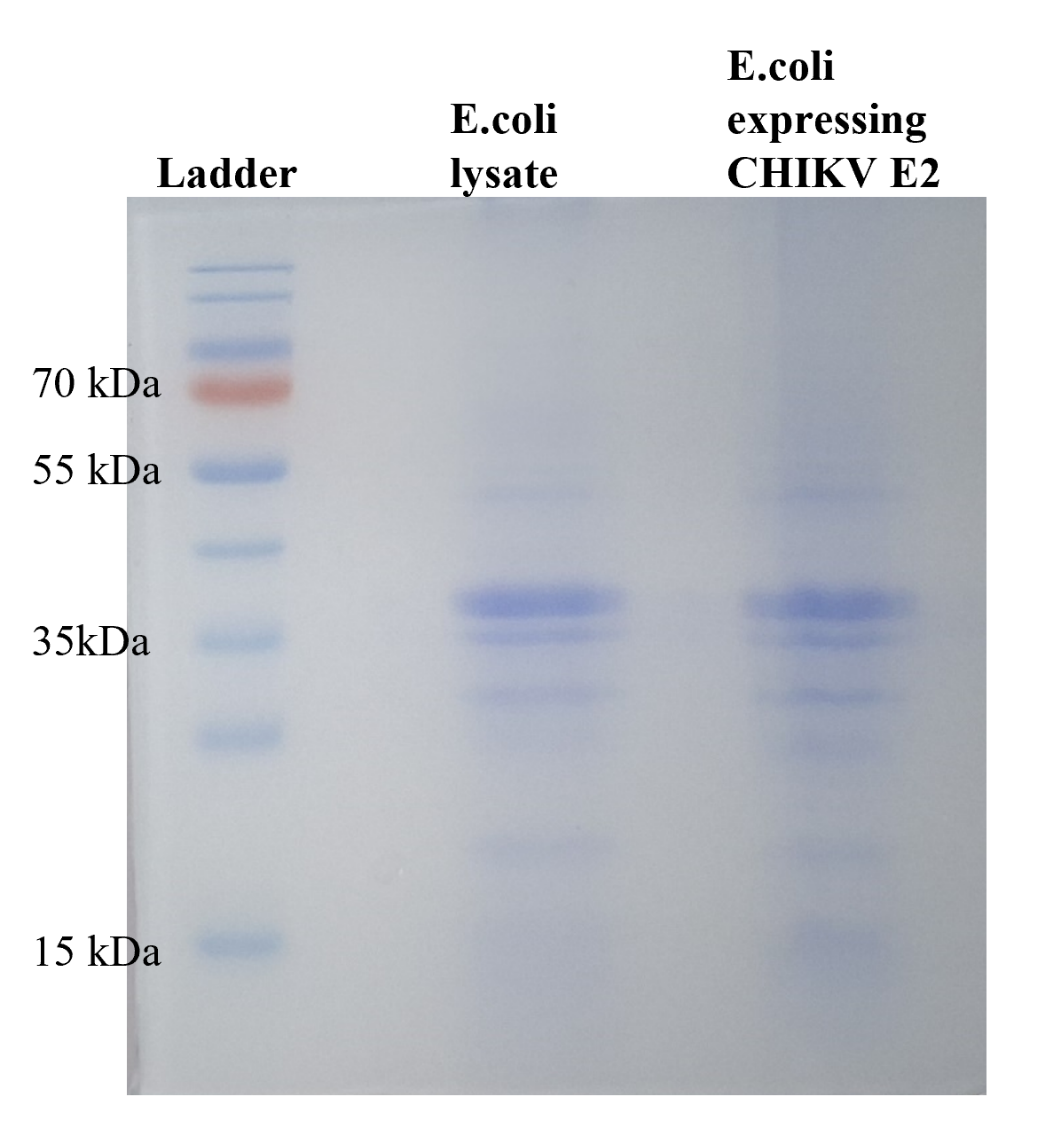


**Fig S3: The bacterial lysate SDS-PAGE image after CBB-staining.**
